# Supplementary material for: Side Chain-Modified Benzothiazinone Derivatives with Anti-Mycobacterial Activity
Source: Biomedicines. 2023 Jul 12;11(7):1975. doi: 10.3390/biomedicines11071975 (PMC10377601; doi:10.3390/biomedicines11071975)
Supplement: Supplementary file 1 [file biomedicines-11-01975-s001.zip › biomedicines-2211257-supplementary.pdf]

Supplementrary Materials

Table of Contents

1. General methods……………………………………………………………………….……S2

2. Synthetic procedures and characterization data for intermediates…….……………….... S2–S6

3. MIC (H37Rv) and DprE1 inhibitory IC_{50} for selected compounds, Table S1…..……..… S7

4. Aqueous Solubility Determination, Table S2……..…………………………….……… S7–S8

5. Compound cytotoxicity assay against HepG2, Figure S1………………..…………..... S8

1. General Methods

Unless otherwise noted, reagents and materials were obtained from commercial suppliers and were used without further purification. Solvents were dried by the appropriate drying agents prior to use. Anhydrous tetrahydrofuran and dichloromethane were obtained from commercial sources. All reactions were monitored by thin layer chromatography (TLC) and column chromatography purification was performed using 300–400 mesh silica gel. Compounds were detected by ultraviolet light (UV)absorption at either 254 or 365 nm. TLC was performed on silica HSGF254 plates. All final products were characterized by ^{1}H NMR,^{13}C NMR and HRMS analyses. ^{1}H NMR and ^{13}C NMR spectra were measured on an Agilent–400 MHz or Bruker DD2-600 MHz spectrometer and referenced to TMS. Analysis of sample purity was performed on SHIMADZU LC-20AD high performance liquid chromatography (HPLC) system. All tested compounds have a purity> 95% before sending for biological test.

Abbreviations: MIC: minimal inhibitory concentration; PEG: poly ethylene glycol; HCl: hydrochloric acid; DCM: dichloromethane; DMF: *N,N*-dimethylformamide; CDI: 1,1'-carbonyldiimidazole; ESI: electrospray ionization; HRMS: high resolution mass spectrometry.

2. Synthetic Procedures and Characterization Data for Intermediates

Preparation of Intermediate 43

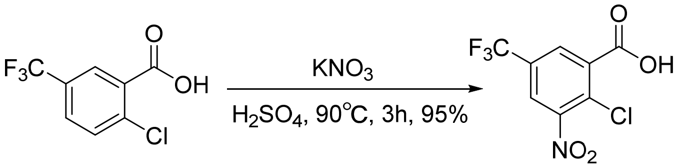

43

To a solution of 2-chloro-5-(trifluoromethyl)benzoic acid (5 g, 22.3 mmol) in 60 mL sulfuric acid was added potassium nitrate (3.38 g, 33.4 mmol) at 0°C. The mixture was then warmed to 90°C. After stirred for 3 h, the resulting solution was cooled to ambient temperature and poured into crushed ice (1.5 g), rinsed with icy water (150 mL). The white precipitate was filtered, washed with cold water, and dried. Yield: 5.7 g (95%).

General synthetic route of benzothiazinone derivatives 45

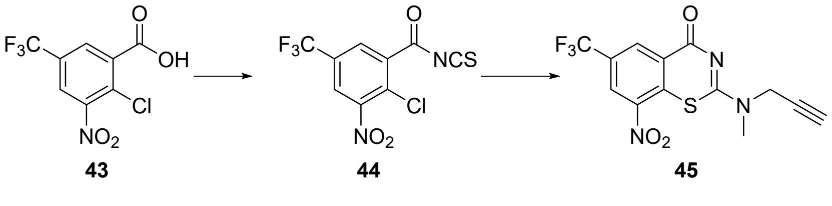

43 **44** **45**

Oxalyl chloride (1.9 g, 14.8 mmol, 4.0 equiv) and catalytic amount of *N,N*-dimethylformamide (DMF) were added successively to the suspension of intermediate **43** (1.0 g, 3.7 mmol, 1.0 equiv.) in 20 mL DCM. The reaction was stirred at room temperature for 2 h and evaporated in vacuo. The residue was redissolved in 20 mL DCM and added to a catalytic amount of PEG-400 under stirring. To which was added ammonium thiocyanate (0.7 g, 9.3 mmol, 2.5 equiv.) in acetone (8 mL) dropwise. The resulting solution was then stirred at room temperature for 0.5 h until the starting materials were consumed, as determined by TLC to get compound **44**. Then *N*-methylprop-2-yn-1-amine (0.3 g, 4.5 mmol, 1.2 equiv.) in 10 mL DCM was added dropwise, and the mixture was stirred for another 2 h and concentrated in vacuo. The residue was then diluted with water (30 mL) and extracted with DCM (3 x 50 mL). The organic extracts were dried over anhydrous Na_2SO_4 and concentrated under reduced pressure. The crude compound was purified by column chromatography using DCM and MeOH (200:1) to give pure intermediate **45** (0.8 g, 60%). ^{1}H NMR (400 MHz, $CDCl_3$) δ 9.13 (s, 1H), 8.79 (s, 1H), 4.76 (s, 2H), 3.64 (s, 1H), 3.45 (s, 3H).

Preparation of aromatic azides

A solution of aromatic amines (2.2 mmol) in CH_3CN (8 mL) was cooled to 0°C in an ice bath. To this mixture was added *t*-BuONO (2.6 mmol) followed by TMS-N_3 (2.58 mmol) dropwise. The resulting solution was stirred at room temperature for 2 h and then concentrated under vacuum, the crude product was purified by silica gel chromatography (hexane) to give the azides in quantitative yield.

Preparation of benzylic and aliphatic azides

To a solution of benzylic/aliphatic halides (4.0 mmol) in THF (10 mL) was added NaN_3 (8.0 mmol) in water (1.0 mL). The resulting solution was stirred at 80 °C for 3 h. The reaction mixture was diluted with EtOAc (20 mL) and washed with water (20 mL) and brine (20 mL). The organic layer was separated, dried (Na_2SO_4) and concentrated under vacuum to give the azides in quantitative yield.

General Procedure for the Synthesis of Compound 1, 2, 4–30

To a stirred solution of compound **45** (0.014 mmol, 1.0 eq) and corresponding azides (0.021 mmol, 1.5 eq) in EtOH (12 mL) under an atmosphere of nitrogen was added a solution of $CuSO_4$ (0.0021 mmol, 0.15 equiv), (+) sodium L-asorbate (0.028 mmol, 0.25 equiv) and K_2CO_3 (0.014 mmol, 1 equiv) in H_2O (4 mL). The mixture was stirred at room temperature for 5 h, and then concentrated under reduced pressure to give the crude residue, which was then partitioned between 10 mL H_2O and DCM 10 mL, and organic phase was washed with brine, dried over anhydrous Na_2SO_4, and filtered, and concentrated under reduced pressure to give the crude product. Purification by flash chromatography (200:1–100:1 CH_2Cl_2–MeOH) afforded the desired 1,2,3-triazol products **1, 2, 4-30**.

General procedure for the preparation of compounds 49

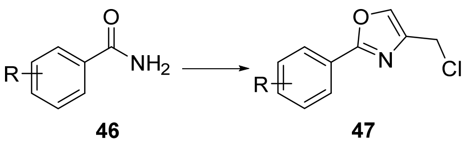

46 47

A mixture of amide 46 (2.5 mmol) and 1,3-dichloroacetone (5.0 mmol) was heated at 130 °C for 1 h. After the mixture was cooled to room temperature, water (20 mL) was added, and the mixture was extracted with DCM (3 × 20 mL). The combined organic layer was dried over anhydrous Na_2SO_4, filtered, and concentrated under reduced pressure, and then the crude product was purified by silica gel column chromatography to afford the corresponding oxazole **47**.

General procedure for the preparation of compounds 48

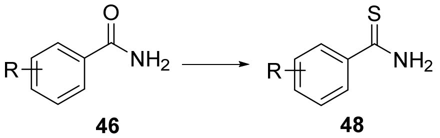

46 48

To a solution of benzamides **46** (2.0 mmol) in dry THF (20 mL) was added Lawesson's Reagent (2.0 mmol). The mixture was stirred at 65 °C for 3 h and then was cooled, concentrated under reduced pressure. The crude product was purified by silica gel column chromatography give the thioamides **48,** yield: 87%-92%.

General procedure for the preparation of compounds 49

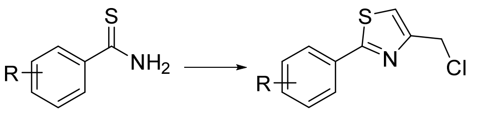

48 **49**

To the thiobenzamides **48** (3.0 mmol) in EtOH (14 mL) and THF (6 mL) was added 1,3-dichloroacetone (3.3 mmol, 1.1 equiv.), and the solution was stirred at 65 °Cfor 12 h. The mixture was cooled and evaporated, and the residue was taken up with EtOAc (15 mL), and washed with an aqueous solution of $NaHCO_3$. The organic phase was dried, filtered and evaporated to afford the thiazole **49**, which was used for the next step without further purification.

General procedure for the preparation of compounds 50a–50c (51a–51c)

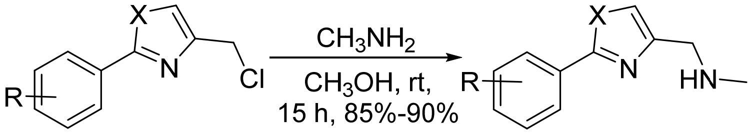

X = "O": **50a**: R = H; **50b**: R = 4-F; **50c**: R = 3-F
X = "S": **51a**: R = H; **51b**: R = 3-F; **51c**: R = 4-F

To a solution of oxazole **47** or thiazole **49** (1.0 mmol) in MeOH (10 mL) was added dropwise a solution of 40% methylamine in MeOH (5 mL) at 0°C. The mixture was stirred for 15 h at room temperature. The reaction was then quenched with 20 mL water, and the mixture was extracted with EtOAc (2 × 20 mL). The combined organic phase was washed with brine, dried over Na_2SO_4, filtered, and concentrated. The residue was purified by silica gel column chromatography to give **50a-50c** or **51a-51c**.

General procedure for the preparation of target compounds 31–36

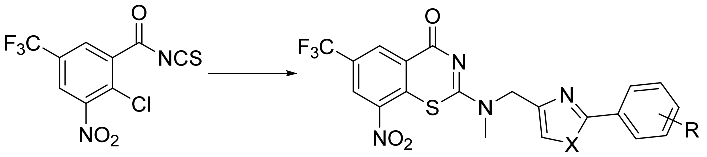

44 **Compound 31-36**

31: X=O, R=H
32: X=O, R=4-F
33: X=O, R=3-F
34: X=S, R=H
35: X=S, R=3-F
36: X=S, R=4-F

Compounds 31–36 were prepared via a similar sequence as described for benzothiazinone derivatives 45, yield 42%–49%. The compound characterization data for final compounds **31**–**36** are shown in the main manuscript.

Preparation of intermediate 52

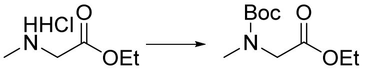

52

Table 500. mg, 3.26 mmol) in DCM (50 mL) was added $(Boc)_2O$ (853 mg, 3.90 mmol) and Et_3N (362 mg, 3.68 mmol) at 0 °C. The resulting solution was stirred at 0 °C for 30 min, and then stirred at room temperature for 12 h. After concentration under reduced pressure, the crude mixture was dissolved in 50 mL DCM and washed with a saturated aqueous solution of NH_4Cl (30 mL), brine (25 mL), dried over anhydrous Na_2SO_4, filtered and concentrated under reduced pressure. Purification by flash chromatography (20 to 30% EtOAc in hexanes) gave **52** as a colorless oil. yield: 650 mg, 92%.

Preparation of intermediate 53

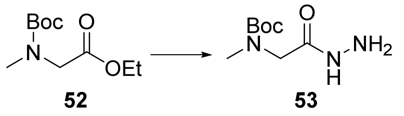

Table 52. (500 mg, 2.30 mmol) in EtOH (25 mL) was added hydrazine hydrate (1.0 mL, 23 mmol, 10.0 equiv.). The resulting solution was refluxed for 5 h and then cooled to ambient temperature. The solvent EtOH was removed in vacuo and the crude product was purified by silica gel chromatography (20 to 30% MeOH in $C_2H_2Cl_2$) to give **53**. yield: 400 mg, 86 %; MS (+ESI): calcd for $C_3H_{10}N_3O$ $[M\text{-}Boc+H]^+$= 104.07, found 104.0.

General procedure for the preparation of compounds 54

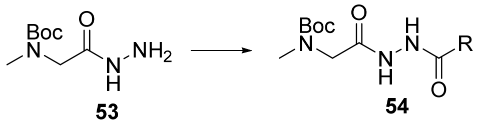

Table 0. mmol) in acetonitrile (10 mL) was added CDI (0.90 mmol). After stirring at 60 °C for 1.5 h, compound **53** (0.82 mmol) was added. The reaction mixture was stirred overnight at room temperature and then concentrated under reduced pressure. The crude product was purified by silica gel chromatography (50 to 90% EtOAc in hexanes) to give **54** in 85-95% yield.

General procedure for the preparation of oxadiazoles 55

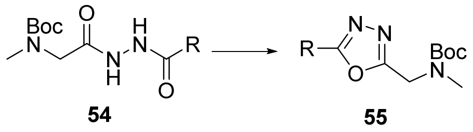

To a solution of **54** (0.70 mmol) in DCM (10 mL) was added imidazole (1.41 mmol), PPh_3 (1.41 mmol) and CBr_4 (1.41 mmol) at 0°C. The reaction was stirred to completion at room temperature. The mixture was then concentrated under reduced pressure, and the crude product was purified by column chromatography (10 to 40% EtOAc in hexanes) to afford oxadiazole **55** (yield 85-90%).

General procedure for the preparation of thiodiazoles 57

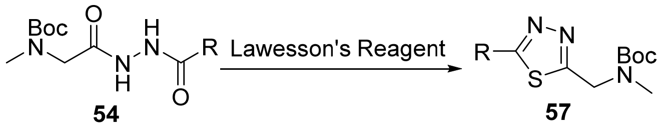

To a solution of compound **54** (2.0 mmol) in dry THF (20 mL) and was added Lawesson's Reagent (2.0 mmol), and the reaction mixture was heated at 65 °C for 3 h, after which the reaction was cooled, concentrated under reduced pressure, and the crude product was purified by silica gel column chromatography to give the cyclization product thiadiazoles **57**, yield 80-90%.

General procedure for the preparation of compounds 56a–c and 58a–c.

To a solution of **55** or **57** (0.70 mmol) in DCM (10 mL) was added dropwise trifluoroacetic acid (4 mL) at room temperature. The reaction mixture was stirred at for 3 h. After the reaction was complete, the mixture was concentrated under reduced pressure to afford **56a-c** (from **55**) or **58a-c** (from **57**) in quantitative yield, which was used for the next step without further purification.

General procedure for the preparation of target compounds 37–42

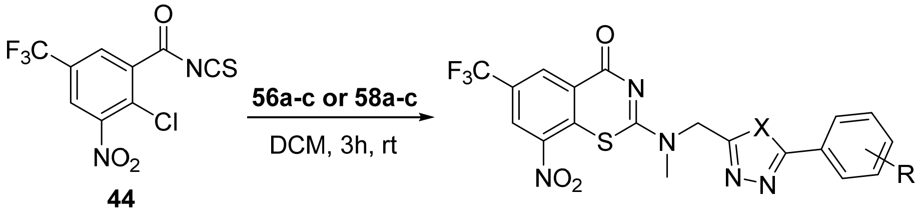

44

37: X=O, R=H
38: X=O, R=3-F
39: X=O, R=4-F
40: X=S, R=H
41: X=S, R=3-F
42: X=S, R=4-F

Compounds **37**–**42** were prepared via a similar procedure as that described for benzothiazinone derivatives **44**, yield: 42%-50%. The characteristic data for final compounds **37**–**42** are shown in the main manuscript.

3. MIC (H37Rv) and DprE1 Inhibitory IC_{50} for Selected Compounds

Table S1. DprE1 inhibitory IC_{50} for selected compounds.

Compound	Structure	MIC, ng/mL	DprE1, IC50 (μM)
**26**	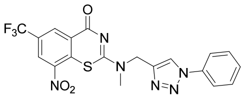	4	0.020
**27**	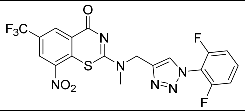	129	7.2
**28**	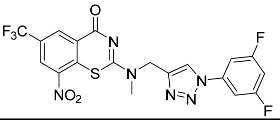	8	0.041
**29**	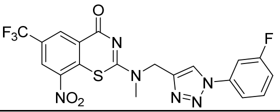	4	0.024
**30**	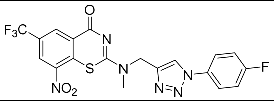	6	0.090
**31**	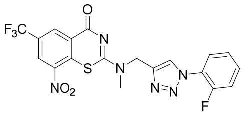	13	0.22
**32**	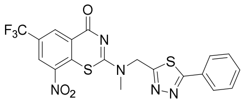	16	0.75
**33**	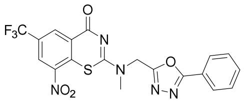	3	0.074
**34**	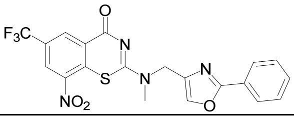	3	0.069
**35**	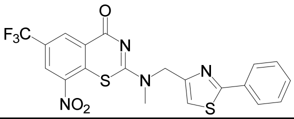	7	0.065
	PBTZ169	0.2	0.009

4. Aqueous Solubility Determination

Kinetic solubility was measured at pH 7.4, test compounds **2** and **37** were initially dissolved in DMSO to make a 10 mM stock solution, the phosphate buffer saline solution contains 45 mM KH2PO4, 45 mM KOAc, 75 mM KCl, 45 mM ethanolamine, pH 7.4. In a total volume of 400 μL with a final DMSO concentration of 1%. A total of 4 μL of stock solution of

compounds at different concentration were mixed with 396 μL of the buffer in a microplate, the solution was incubated and shaken for 4 h at room temperature. The mixture was then filtered through a 0.3 μm pore size membrane filter. The filtrate was diluted by 10× and 30× PBS buffer and vortexed. The diluted solutions were analyzed by LC-MS/MS. A series of calibration compound solutions was prepared to generate a standard curve.

Table S2. Kinetic solubility of compounds **2** and **37** in 50 mM PBS at pH 7.2.

	**2**	**37**	**PBTZ169**
Solubility (μg/mL)	0.13	0.25	<0.01[10]
clogP *	4.04	3.04	5.10

* clogP is calculated using ChemBioDraw 14.0.

5. Cytotoxicity assay

HepG2 cells were obtained from the Chinese Type Culture Collection (Shanghai, China) and maintained in Dulbecco's modified eagle medium (Gibco; #C11995500BT) containing 1% (*v*/*v*) penicillin/streptomycin (Beyotime; #C0222) and 10% (*v*/*v*) fetal bovine serum (Gibco; #GB10270106) in an atmosphere of 5% CO_2 at 37 °C. HepG2 cells were seeded into 96-well plates at a density of 5,000 cells/well (100 μL total volume) and treated with different concentrations of compound **2** and **37** (0, 0.025, 0.2, 1, 5, and 10 μg/mL). After 24 h the medium was removed and the cells were incubated in a fresh medium containing 5 mg/mL MTT for 4 h. The medium was then removed and the precipitated formazan was dissolved in 100 μL of DMSO followed by shaking for 10 min. Finally, the absorbance at 570 nm was measured on a microplate spectrophotometer. Each treatment was performed in triplicate.

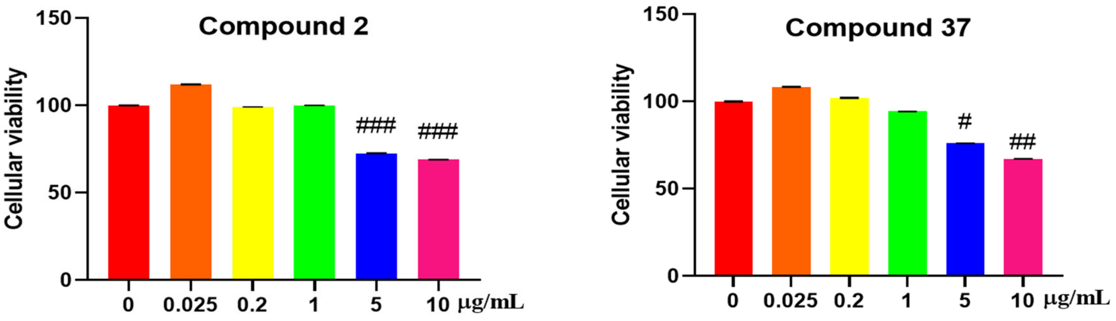

Figure S1. Inhibitory activity of compounds **2** and **37** against HepG2 cell.
